# Oscillations of Cerium Oxidation State Driven by Oxygen Diffusion in Colloidal Nanoceria (CeO_2 − *x*_)

**DOI:** 10.1186/s11671-017-2339-7

**Published:** 2017-10-13

**Authors:** Yuri Malyukin, Vladimir Klochkov, Pavel Maksimchuk, Vladyslav Seminko, Nikolai Spivak

**Affiliations:** 1 Institute for Scintillation Materials, National Academy of Sciences of Ukraine, 60 Nauky Ave, Kharkiv, 61001 Ukraine; 2 Zabolotny Institute of Microbiology and Virology, National Academy of Sciences of Ukraine, 154 Akademika Zabolotnogo St, Kyiv, 03680 Ukraine

**Keywords:** Oxygen vacancies, Clusters, Luminescence, Antioxidants

## Abstract

The redox performance of CeO_2 − *x*_ nanocrystals (nanoceria) is always accompanied by the switching of cerium oxidation state between Ce^3+^ and Ce^4+^. We monitored Ce^3+^ → Ce^4+^ oxidation of nanoceria stimulated by oxidant in aqueous colloidal solutions controlling the luminescence of Ce^3+^ ions located at different distances from nanoceria surface. The observed Ce^3+^ luminescence changes indicate that Ce^3+^ → Ce^4+^ reaction develops inside nanoceria being triggered by the diffusing oxygen originated from the water splitting on oxidized nanoceria surface. We present the first observation of the pronounced oscillations of Ce^3+^ luminescence intensity arising from Ce^3+^ ↔ Ce^4+^ reversible switching. This threshold effect is to be driven by uptaking and releasing oxygen by nanoceria, when the concentration of oxygen vacancies in nanoceria lattice, oxidant concentration in colloidal solution, and temperature reach certain critical values. So, the ability of nanoceria to uptake and release oxygen depending on the environmental redox conditions really makes it the self-sufficient eternal antioxidant.

**Electronic supplementary material:**

The online version of this article (10.1186/s11671-017-2339-7) contains supplementary material, which is available to authorized users.

## Background

Today, nanocrystals with different structure and chemical composition are widely used in great diversity of modern applications [1–9]. Along with important engineering utilizations [3, 4], CeO_2_ nanocrystals (nanoceria) gave birth to promising biomedical developments [5–9] owing to its ability to work as a regenerative scavenger of reactive oxygen species (ROS). The main prerequisite that makes nanoceria so unique and useful is generally attributed to high content of oxygen vacancies (V_O_) and Ce^3+^ ions on its surface [10–14]. In nanoceria lattice, V_O_ and Ce^3+^ ions are interrelated defects [10–14]; two Ce^3+^ ions are accounted for one V_O_ [13]. The defect (Ce^3+^, V_O_) concentration in nanoceria can be controlled by particle size, special doping, and temperature treatment [11, 14, 15]. In general, the surface oxygen can assist the redox cycle through V_O_ creation and healing or surface V_O_ can act as binding sites for catalytically active species [3, 4, 14]. The surface Ce^3+^ ions of nanoceria are commonly supposed to provide ROS scavenging due to switching between 3+ and 4+ oxidation states [5–9]. ROS, namely superoxide ions ${\mathrm{O}}_2^{-}$, hydroxyl radicals OH˙, and hydrogen peroxide H_2_O_2_ at low concentrations, are critically important for the regulation of cell functions [5–9]. Unlike ordinary antioxidants, which disappear irretrievably after interaction with ROS [5–7], nanoceria, at particle sizes below 15 nm, can act as a self-regenerating antioxidant [5–9]. The critical dependence of nanoceria biological activity on its size, as well as the self-regeneration mechanism of nanoceria in biological environment, is still poorly understood [5–7], and discussions are continuing [8, 9]. It should be stressed that in in vitro and in vivo experiments [5–9], the nanoceria operate at a high defect concentration and water activity and its redox performance can be strongly masked by the cell antioxidant systems.

So, to understand the mechanism of nanoceria redox performance, we used more simple and controlled conditions: the nanoceria oxidation dynamics was studied in aqueous colloidal solutions for nanoceria specimens with variation of oxygen deficiency. As V_O_ (Ce^3+^) concentration vary with the particle size [10–12], the nanoceria specimens of 3.0, 10.0, and 50.0 nm were used. According to the data [12], V_O_ concentration in 3.0-nm nanoceria can reach up to ~ 20%. We determined that V_O_ concentration in 10.0-nm nanoceria, as compared with 3.0-nm nanoceria, was twice less (see Additional file 1). In the case of 50.0-nm nanoceria, doping with Y^3+^ (or Eu^3+^) ions and vacuum annealing were used to generate V_O_ and to create different conditions for oxygen diffusion in nanoceria lattice [14, 15]. In all 50.0 nm samples, V_O_ concentration was made equivalent to 10.0-nm nanoceria. At Re^3+^ doping of 50.0-nm nanoceria, the concentrations of Y^3+^ and Eu^3+^ ions were at the level of 10 at.% (see Additional file 1). These concentrations were sufficiently lower than the values of corresponding solubility limits for these ions in ceria lattice, ~ 25 at.% [16] or even ~ 45 at.% [17] for Y^3+^ ions and ~ 30 at.% [18] for Eu^3+^ ions; so, formation of Y_2_O_3_ or Eu_2_O_3_ phases can be excluded. All colloidal solutions contained the same amount of the substance (1.0 g/l) and were characterized by initial pH ~ 7. For nanoceria oxidation, hydrogen peroxide (HP) and potassium periodate KIO_4_ (PP) were used. PP allowed us to exclude the chemical diversity of HP. The details of the nanoceria synthesis and characterization of the obtained specimens, as well as a description of the experiments, are presented in the Additional file 1.

## Results and Discussion

**Fig. 1:**
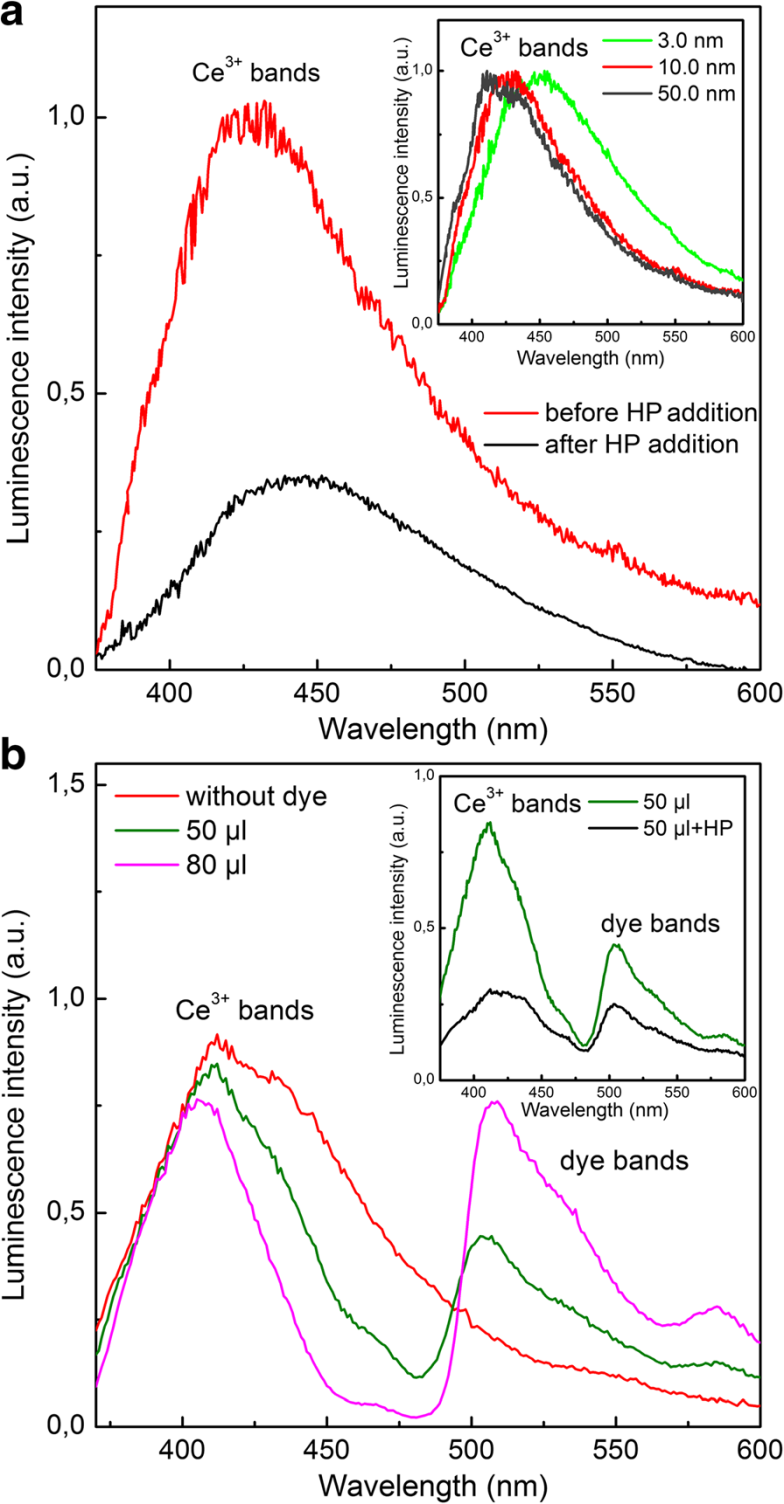
Luminescence spectra of nanoceria under different conditions. **a** 10.0-nm nanoceria before and 25 min after HP (*C* = 0.1 mM) addition. Insert: normalized spectra of different nanoceria samples. **b** Luminescence spectra of 10.0-nm nanoceria before and after addition of 50 and 80 μl of dye. Insert: spectra of 10.0-nm nanoceria after addition of 50 μl of dye and subsequent addition of HP (*C* = 0.1 mM)

Relying on our preliminary experiment [19], the Ce^3+^ luminescence (Fig. 1a) due to the dipole-allowed 5d → 4f optical transitions of Ce^3+^ ions [20] was used to monitor the oxidation dynamics of all tested nanoceria specimens. The increase of the Ce^3+^ band asymmetry with the decrease of particle size, that is, with the increase of the surface-to-bulk ratio (see insert in Fig. 1a) indicates clearly its inhomogeneity. The long-wave part of this band can be attributed to the Ce^3+^ luminescence from the nanoceria subsurface layer, and the remaining part of the Ce^3+^ band comes from deep-seated Ce^3+^ ions. The subsurface Ce^3+^ ions have the red-shifted luminescence spectra (see insert in Fig. 1a) because of the weakening of the crystal field acting on these ions as a result of the lattice parameter increase in the direction to the nanoceria surface [12]. Contrary to 3.0-nm nanoceria, for 50.0-nm nanoceria, the impact of these ions to the resulting luminescence spectrum is negligible (see insert in Fig. 1a). This attribution was confirmed by the stronger Forster quenching [21] of the long-wave part of the Ce^3+^ band (see Additional file 1 and Fig. 1b). The increase of the quencher concentration resulted in the luminescence quenching of deeper lying Ce^3+^ ions (Fig. 1b) because of the reduction of the donor-acceptor distance [21].

**Fig. 2:**
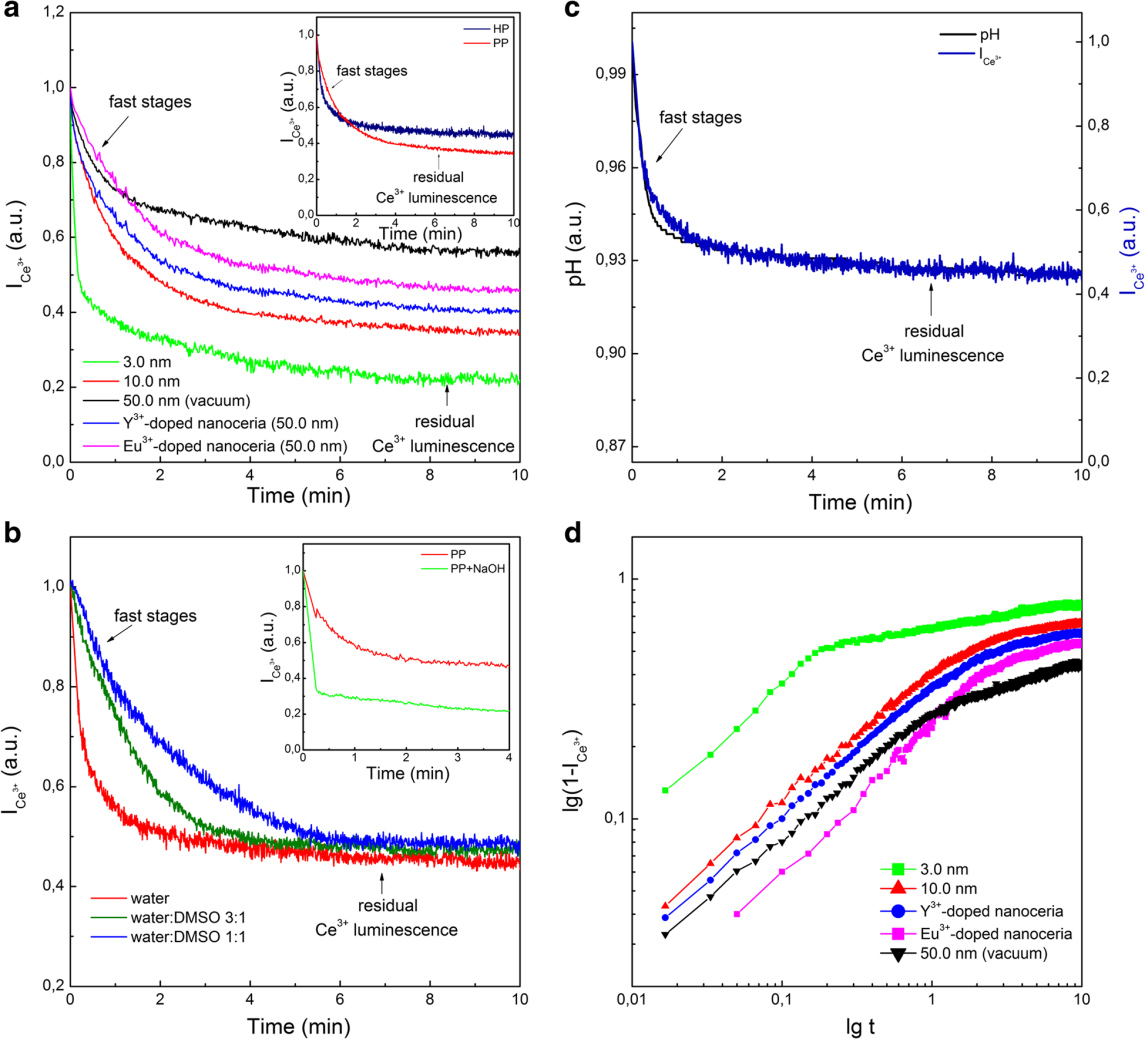
Time evolution of Ce^3+^ luminescence under the oxidant action (*C* = 0.1 mM) for different specimens of nanoceria. **a** After HP addition; insert—after HP and PP addition; **b** after PP addition to 10.0-nm nanoceria in water-DMSO solutions; insert—after PP addition and after NaOH and subsequent PP addition. **c** pH and Ce^3+^ band intensity (${I}_{{\mathrm{Ce}}^{3+}}$) after PP addition for 10.0-nm nanoceria. **d** Curves of nanoceria filling by oxygen *ρ*(*t*)=1−${I}_{{\mathrm{Ce}}^{3+}}(t)$ shown on a log-log plot using data shown in **a**

Adding oxidant (HP or PP) to colloidal solutions resulted in a decrease of the Ce^3+^ band intensity for all tested nanoceria specimens (Fig. 1a). Moreover, one can see that under selective quenching of the Ce^3+^ band (Fig. 1b), the oxidant stimulated a drop in luminescence of the deep-seated Ce^3+^ ions (see insert in Fig. 1b). The Ce^3+^ → Ce^4+^ oxidation occurs for these ions as well in spite of the fact that the oxidant cannot penetrate into nanoceria. This effect is similar to annealing nanoceria in oxygen atmosphere (see Additional file 1). Hence, the oxidant stimulates the penetration of oxygen (its source will be determined below) inside nanoceria. It is really corroborated by the defect-controlled time evolution of Ce^3+^ luminescence under oxidant action (Fig. 2a). In this experiment, both HP and PP act in a similar way (see insert in Fig. 2a). As follows from Fig. 2a, the 3.0-nm nanoceria with the highest V_O_ concentration demonstrated the fastest drop of the Ce^3+^ band intensity and the lowest residual Ce^3+^ luminescence. At the same V_O_ concentration, the Y^3+^-doped nanoceria showed a stronger quenching rate of the Ce^3+^ band intensity and lower level of residual Ce^3+^ luminescence as compared to the Eu^3+^-doped nanoceria (Fig. 2a). It correlates with the fact that the activation energy of oxygen diffusion in cerium oxide increases in the presence of Eu^3+^ ions in a stronger way than of Y^3+^ ions [14, 15]. Hence, the Ce^3+^ band intensity decrease under oxidant action (Figs. 1 and 2a) is a result of Ce^3+^ → Ce^4+^ oxidation caused by oxygen penetration into nanoceria via the vacancy mechanism of diffusion [14, 15]. The addition of the reducing agent (for example, benzenetriol) to the colloidal solution upon nanoceria oxidation did not lead to the Ce^3+^ luminescence recovery, which is consistent with the proposed mechanism of Ce^3+^ → Ce^4+^ oxidation inside nanoceria.

**Fig. 3:**
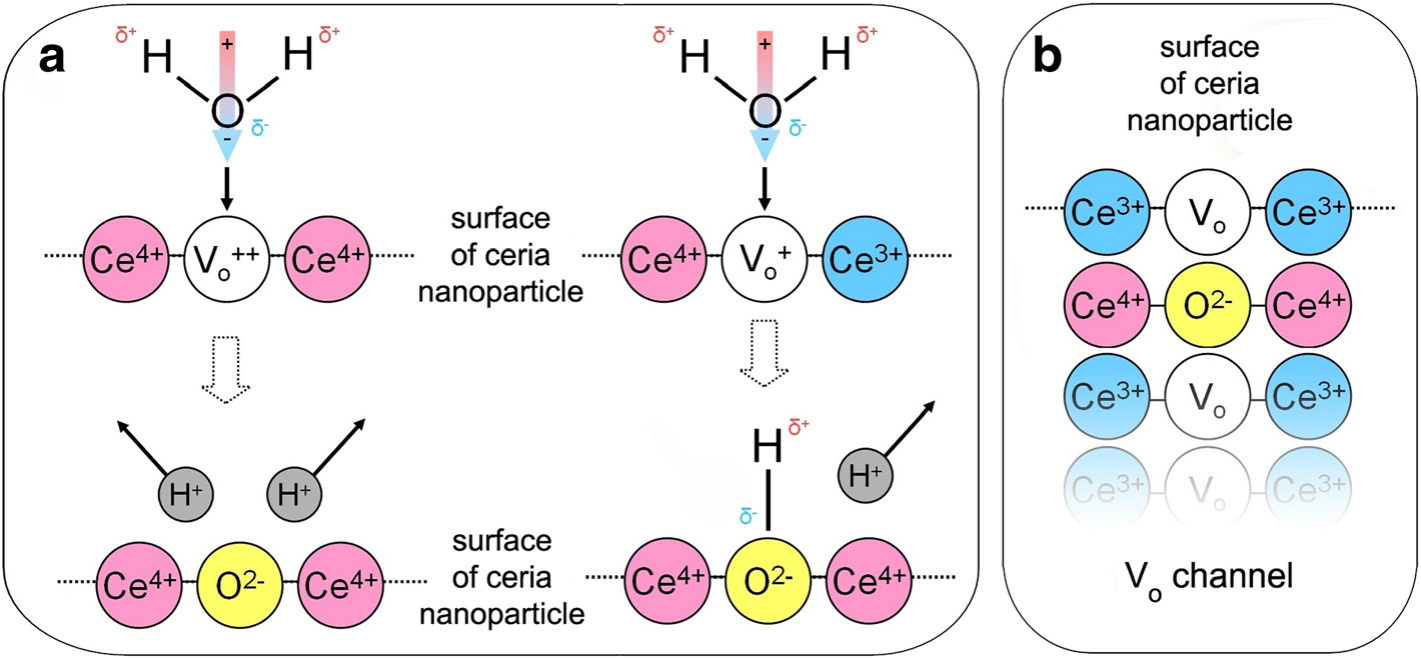
The stages of the nanoceria interaction with oxidant and water molecule. **a** Double-oxidized Ce^4+^–${\mathrm{V}}_{\mathrm{O}}^{++}$–Ce^4+^ site and single-oxidized Ce^4+^–${\mathrm{V}}_{\mathrm{O}}^{+}$–Ce^3+^ site on nanoceria surface and their interaction with H_2_O molecule. **b** Regeneration of Ce^3+^–V_O_–Ce^3+^ site for next oxidation cycle

The lower water concentration in the colloidal solution slowed down the dynamics of nanoceria oxidation (Fig. 2b), while the increase of initial pH accelerated this process (see insert in Fig. 2b). Excluding possible source of H^+^ in the case of HP application, we have also revealed the exact coincidence of the pH decrease with the Ce^3+^ band intensity drop under the nanoceria oxidation by PP (Fig. 2c). These facts indicate the water splitting, which can proceed with high efficiency with participation of the Ce^4+^ $\hbox{--} {\mathrm{V}}_{\mathrm{O}}^{++}$–Ce^4+^ (or Ce^4+^ $\hbox{--} {\mathrm{V}}_{\mathrm{O}}^{+}$–Ce^3+^) active sites on the nanoceria surface forming as a result of the oxidation of Ce^3+^-V_O_-Ce^3+^ sites (Fig. 3) [13]. There are two possible ways for that (Fig. 3): either the O^2−^ ion occupies ${\mathrm{V}}_{\mathrm{O}}^{++}$ and two H^+^ ions are ejected to the solution or the O^2−^ ion occupies ${\mathrm{V}}_{\mathrm{O}}^{+}$ resulting in the creation of hydroxyl, which makes one H^+^ ion to be ejected to solution (Fig. 3). The first jump of oxygen into nanoceria regenerates the Ce^3+^–V_O_–Ce^3+^ site for a new oxidation cycle (Fig. 3). This process can be repeated many times with different rates depending on oxidant concentration. The curve $\uprho (t)=1-{I}_{{\mathrm{Ce}}^{3+}}(t)$ describes the filling of nanoceria with oxygen, and its initial stage fits well the ~*t* ^1/2^ function (see Fig. 2d). It means that oxygen penetrates into the nanoceria by the single-file diffusion through V_O_ channels (Fig. 3), where oxygen atoms cannot bypass each other [22, 23]. The formation of large V_O_ clusters opened onto the oxygen-terminated planes of nanoceria (Fig. 3) is unavoidable for two reasons: all tested nanoceria specimens contain high enough V_O_ concentrations close to the percolation threshold [24] and V_O_ concentration reaches its maximum value near the nanoceria surface [10–12]. The linear structures [25, 26] observed for the subsurface V_O_ may be considered as V_O_ channels or as components of large V_O_ clusters.

**Fig. 4:**
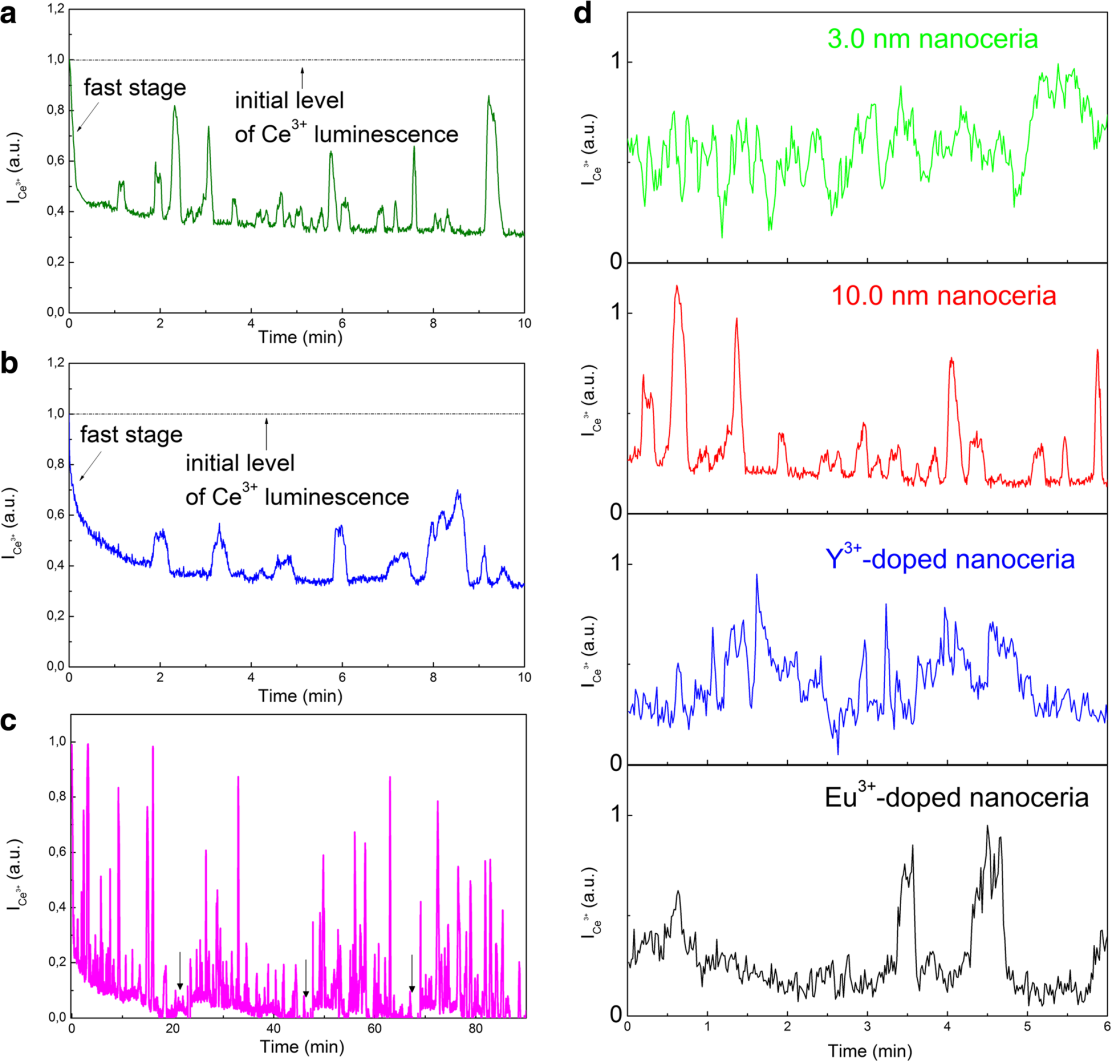
The oscillations of Ce^3+^ band intensity (${I}_{{\mathrm{Ce}}^{3+}}$) stimulated by oxidant (*C* = 1.0 mM) in colloidal nanoceria: **a** after HP addition for 10.0-nm nanoceria; **b** after PP addition for 10.0-nm nanoceria; **c** after multiple HP addition for 10.0-nm nanoceria; **d** after HP addition for different nanoceria samples

The pronounced oscillations of the Ce^3+^ band intensity are observed when the oxidant (HP or PP) concentration in colloidal solutions exceeds ~ 0.5 mM, so that the dynamics of nanoceria Ce^3+^ → Ce^4+^ oxidation transform to Ce^3+^ ↔ Ce^4+^ redox scenario (Fig. 4). These oscillations did not appear immediately after the HP (*C* = 1.0 mM) or PP (*C* = 1.0 mM) addition but started to develop when the fast stage comes to completion (Fig. 4a, b). For all oxidants, after the oscillations disappeared, they could be repeated by adding new portions of the oxidant to the colloidal solutions (Fig. 4c). For comparison, the oscillations of the Ce^3+^ band intensity for all tested nanoceria specimens are shown in Fig. 4d. The base lines in Fig. 4d are actually the levels of the residual Ce^3+^ luminescence for each nanoceria specimen (Fig. 2a), and the Ce^3+^ band intensity oscillates above these levels. The variation of V_O_ concentration in the Y^3+^ (or Eu^3+^)-doped 50.0-nm nanoceria showed clearly that the oscillations of the Ce^3+^ band intensity were observable only when the V_O_ concentrations became equivalent to those in the 10.0-nm nanoceria (Fig. 4d). In the case of Eu^3+^-doped 50.0-nm nanoceria the oscillations were more irregular and less pronounced (Fig. 4d). As it was mentioned earlier, this fact is consistent with the suppression of oxygen diffusion in the presence of Eu^3+^ ions [14, 15]. In the annealed 50.0-nm nanoceria with thermodynamically nonequilibrium V_O_, the oscillations were not observed at all. The oscillations (see Fig. 4) were observable at the temperatures above 35 °C only.

The observed oscillations (Fig. 4) appeared when the population of V_O_ by oxygen accumulated during the fast stage (Figs. 2a and 4a) became strongly nonequilibrium. The growth of the first peak of oscillations (Fig. 4a, b) is accompanied by the release of excess oxygen, and as long as the oscillations are observed, the nanoceria releases (Ce^3+^ luminescence increase) and uptakes (Ce^3+^ luminescence decrease) oxygen. For the annealed 50.0-nm nanoceria, the phase of oxygen release and, hence, the Ce^4+^ ↔ Ce^3+^ oscillations are impossible because the thermodynamically nonequilibrium V_O_ are irreversibly healed by oxygen accumulated during oxidation (Figs. 2a and 4).

It should be noted that the time scale of the Ce^3+^ band intensity evolution (see Figs. 2 and 4) requires an anomalously high rate of oxygen diffusion in nanoceria at RT. Generally, in oxides, the ordinary oxygen diffusion is too slow owing to the large value of the activation energy [14, 15]. But in our case, the fast oxygen diffusion is provided by the loading-dependent reduction of the activation energy inherent for single-file diffusion [23]. The oxidant concentration in the colloidal solutions controls this effect via the rate and level of the filling of V_O_ clusters with oxygen.

## Conclusions

Our results suggest new vision of microscopic mechanisms behind the nanoceria redox performance. First of all, both the surface Ce^3+^ ions available for the oxidant and the deep-seated Ce^3+^ ions are involved in the oxidation dynamics of nanoceria due to oxygen diffusion supported by the open V_O_ clusters. Such V_O_ clusters are inevitably formed at a sufficiently small size (< 15 nm) of nanoceria that explains the strong size dependence of nanoceria antioxidant activity. The self-regeneration (reverse Ce^4+^ → Ce^3+^ reduction) of nanoceria in biological environment is a result of releasing the oxygen accumulated during its oxidation by ROS. Similar to the oscillations in heterogeneous catalysis [27], the oscillations of cerium oxidation state in nanoceria can be exploited for the development of high-performance antioxidants, which are extremely important for cell protection under high-intensity radiation (cancer radio-treatment, nuclear catastrophes, etc.). Overall, the ideas suggested in the paper allow to initiate a rational search for new nanomaterials that can be utilized not only as effective antioxidants, but also as unique catalytic materials in various technological areas.

## Methods

### Methods of Nanoceria Synthesis

#### Colloidal Synthesis of 3.0- and 10.0-nm Nanoceria

Aqueous solutions of ceria nanoparticles were obtained by the following method: CeCl_3_ solution (100 ml, 2 mM) was mixed with 100 ml of hexamethylenetetramine solution (4 mM) and stirred by means of magnetic stirrer for 3 h at room temperature. After that, 1.8 ml NH_4_OH and 0.6 ml of H_2_O_2_ were added into the solution. Then, the solution was put in round-bottom flask and refluxed for 5 h. As a result, transparent colorless solutions were obtained. The solution was evaporated in a rotary evaporator flask under vacuum at the bath temperature of 70 °C to 30 ml. A solution of 2 M NaCl was added to the obtained solution until the resulting solution became turbid. Then, the solid phase was precipitated by centrifugation. The precipitate was separated, and solution of sodium chloride was added again. The procedure of precipitate cleaning was repeated three times. After the last stage of centrifugation, solution of sodium citrate with molar ratio CeO_2_/NaCt of 1:1 was added to the precipitate. Size of nanoceria obtained from the mixture of cerium (III) chloride and hexamethylenetetramine (HMTA) taken in mole ratio 1:10 was ~ 10.0 nm. At further increase of HMTA, excess size of obtained nanoparticles decreases to ~ 3.0 nm. СеО_2 − *х*_ nanoparticles were stabilized by sodium citrate with molar ratio 1:1. The solutions were additionally dialyzed for 24 h against deionized water to remove the excess of ions and organics species. Dialysis membrane tubing with a molecular weight cutoff of “Cellu Sep H1” 3.5 KDa was used. All sols were transparent in transmitted light and passed through membrane filters with pores of 100 nm without loss.

#### Sol–gel Synthesis of 50.0-nm Nanoceria

СеО_2 − *x*_, СеО_2_:Eu^3+^/Y^3+^(0.1–10 at.%) nanocrystals were obtained by Pechini method. Cerium oxide (СеO_2_) (99.995%, Sigma-Aldrich) was dissolved in the mixture of nitric acid (НNO_3_) and hydrogen peroxide (Н_2_О_2_) (in 1:1 volume ratio) at 60 °C. Europium oxide (Eu_2_O_3_) (99.999%, Sigma-Aldrich) and yttrium oxide (Y_2_O_3_) (99.999%, Sigma-Aldrich) were dissolved in the dilute НNO_3_ at 80 °C. The solution of 0.75 g of citric acid and 1 ml of ethylene glycol was added to 20 ml of cerium nitrate Се(NO_3_)_3_ (*C* = 1 M) solution or to 20 ml the stoichiometric mixture of cerium nitrate Се(NO_3_)_3_ (*C* = 1 M) and europium nitrate Eu(NO_3_)_3_/yttrium nitrate Y(NO_3_)_3_ (*C* = 1 M) solutions. All the resulting mixtures were treated at 80 °С during 10 h and then hydrolyzed by means of 10 mass.% NH_3_ aqueous solution. The precipitates were dried at 120 °С during 5 h and then dehydrated at 250 °С during 4 h. The nanocrystals were annealed during 2 h in vacuum at 1000 °C. After annealing, nanoparticles were dispersed in water at 1 g/l concentration.

### Experimental Techniques

The photoluminescence of all types of nanoceria has been excited by a continuous-wave GKL-4UM He-Cd laser (*λ*~325 nm) and registered using the SDL-1 grating monochromator with the Hamamatsu R9110 PMT in the photon-counting mode.

Immediately after oxidant (hydrogen peroxide or potassium periodate) addition to aqueous colloidal solutions of nanoceria, the time evolution of Ce^3+^ luminescence intensity (taken at 390 nm) was determined by means of time-resolved measurements at CW excitation (He–Cd laser). Concentrations of nanoceria in aqueous solutions were similar in all experiments and equal to 1 g/l. Concentration of oxidant required to initiate non-reversible Ce^3+^ → Ce^4+^ redox reaction was equal to 0.1 mM; concentrations of oxidants for initiation of reversible Ce^3+^ ↔ Ce^4+^ redox reactions were equal to 1.0 mM for both H_2_O_2_ (HP) and KIO_4_ (PP).

The time dependence of pH value for the nanoceria aqueous colloidal solutions after oxidant addition was measured using pH meter. As the oxidants HP (*C* = 0.1 mM) and PP (*C* = 0.1 mM) were used, pH values were recorded with time intervals of 1 s after addition of the portions of oxidant to nanoceria aqueous colloidal solutions. The time dependence of pH value for distilled water after addition of the oxidant was taken as a control (initial pH value of distilled water was the same as the one for colloidal solutions of nanoceria (pH = 7)).

All experiments were realized at *T* = 37 °С.

## Additional file

Additional file 1. Supplementary materials. (DOCX 1515 kb)

## Abbreviations

HP: Hydrogen peroxide
PP: Potassium periodate
ROS: Reactive oxygen species
V_O_: Oxygen vacancies
