# Supplementary material for: Oscillations of Cerium Oxidation State Driven by Oxygen Diffusion in Colloidal Nanoceria (CeO2 − x)
Source: Nanoscale Res Lett. 2017 Oct 13;12:566. doi: 10.1186/s11671-017-2339-7 (PMC5640564; doi:10.1186/s11671-017-2339-7)
Supplement: Supplementary file 1 — Supplementary materials. (DOCX 1515 kb) [file 11671_2017_2339_MOESM1_ESM.docx]

**Supporting Information**

Characterization of nanoceria

The size and crystalline phase of all types of nanoceria were verified by TEM (Figure S1) and XRD (Figure S2). According to TEM data, the sizes of nanocrystals were equal to: 3.0±0.5 nm, 10.0±1.5 nm and 50.0±5 nm. In the paper these samples are referred to as 3.0 nm nanoceria, 10.0 nm nanoceria and 50.0 nm nanoceria. X-ray diffraction patterns of obtained СеO_2_ and СеО_2_:Eu^3+^/Y^3+^ nanocrystals corresponds with JCPDS card No.34-0394, so the nanocrystals are characterized by FCC fluorite-type lattice and formation of any additional phases at these conditions can be excluded. Decrease of the size of nanoceria from 50.0 nm to 3.0 nm leads to sufficient widening of XRD peaks due to increase of the lattice parameter of ceria lattice.

ζ-potential of nanoceria in colloidal solutions was measured using ZetaPALS (Brookhaven Instruments Corp., USA) analyzer at scattering angle of 15° in special polystyrene cuvettes (BI-SCP). ζ-potential was calculated using Doppler shift of the beam scattered by nanoparticles with following re-calculation by Smoluchowski equations. All nanoceria samples had negative charge, ζ-potential for 3.0 nm nanoceria was equal to -23.5 mV, for 10.0 nm nanoceria -25.0 mV.

Control of Се^3+^ and V_O_ concentrations in tested nanoceria specimens

Since in nanoceria lattice concentration of Се^3+^ ions is proportional to V_O_ concentration, the comparison of the Се^3+^ band intensities in the luminescence spectra normalized by the CT band intensity (Figure S3) allows the relative concentration of defects in nanoceria specimens to be controlled. It was found that in 3.0 nm nanoceria, concentration of Се^3+^ ions and V_O_ concentration were almost twice as much as compared with 10.0 nm nanoceria (Figure S3). In the rest of the nanoceria specimens specially prepared for experiment, namely Y^3+^ (or Eu^3+^) doped 50.0 nm nanoceria and annealed 50.0 nm nanoceria concentration of Се^3+^ ions and V_O_ concentration were the same as in 10.0 nm nanoceria. The ratio Ω$={I_{{Ce}^{3+}}}/{I_{CT}}$, where $I_{{Ce}^{3+}} \mathrm{and} I_{CT}$ are intensities of luminescence of the Ce^3+^ band and the CT band respectively, has been used as an indicator of nanoceria nonstoichiometry (Ω$=0$ for CeO_2_) to select the nanoceria specimens for the experiments. Thus, four nanoceria specimens have been taken, namely, 10.0 nm nanoceria, Eu^3+^-doped 50.0 nm nanoceria, Y^3+^-doped 50.0 nm nanoceria with Ω~6.0 which had the same concentration of Ce^3+^ ions and 3.0 nm nanoceria with Ω~11.0.

Nanoceria annealing in an oxygen atmosphere was accompanied by oxygen diffusion into nanoceria and leads to the same decrease of Се^3+^ band intensity as oxidant addition (Figure S4), so the latter can be ascribed to oxygen diffusion into nanoceria lattice as well.

Quenching of nanoceria luminescence by dye molecules

In the experiments with quenching of Ce^3+^ luminescence by dye molecules two cyanine dyes were used: DiOC2 cationic dye and DiOC3S anionic dye. In the Figure S5 the structures of both dyes are shown. Use of both cationic and anionic dyes was necessary in order to exclude formation of complexes between nanoceria particles and dye molecules that would lead to luminescence quenching via electron transfer but not via energy transfer. Absorption spectra of DiOC2 and DIOC3S are similar and have a sufficient overlap with luminescence spectrum of nanoceria (Figure S6), so an effective energy transfer between nanoceria particles and dye molecules must be observed. The overlap integrals J(λ) are equal to ~6.4⋅10^-14^ for both DiOC2 and DIOC3S. Förster radius was determined as $R=9.78\cdot10^{3}\left(\frac{k^{2}Q_{D}J(\lambda)}{n^{4}}\right)^{1/6}$), where Q_D_ is the quantum yield of donor luminescence, n is refractive index. As the quantum yield of CeO_2_ luminescence cannot be estimated with precise accuracy, Förster radius for energy transfer between CeO_2_ and molecules of both dyes roughly falls into an interval of 1.5 - 2.0 nm.

Transformations of nanoceria luminescence spectra after addition of DiOC3S anionic dye or DiOC2 cationic dye are shown in Figure S7 and Figure S8, respectively. Nanoceria is negatively charged, so cationic dye molecules must be closer to ceria surface as compared to anionic dye molecules. As was expected, the increase of the average distance resulted in a weakening of the quenching effect, and simultaneously in a narrowing of the spectral part of the Ce^3+^ band which was quenched.

**Figures S1-S8:**

**
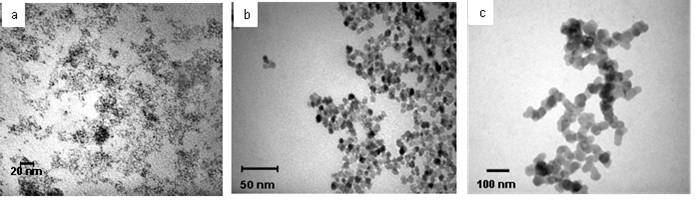
**

**Figure S1.** ТЕМ of tested nanoceria specimens: **a,** 3.0 nm nanoceria; **b,** 10.0 nm nanoceria and **c,** 50.0 nm nanoceria as obtained.

**
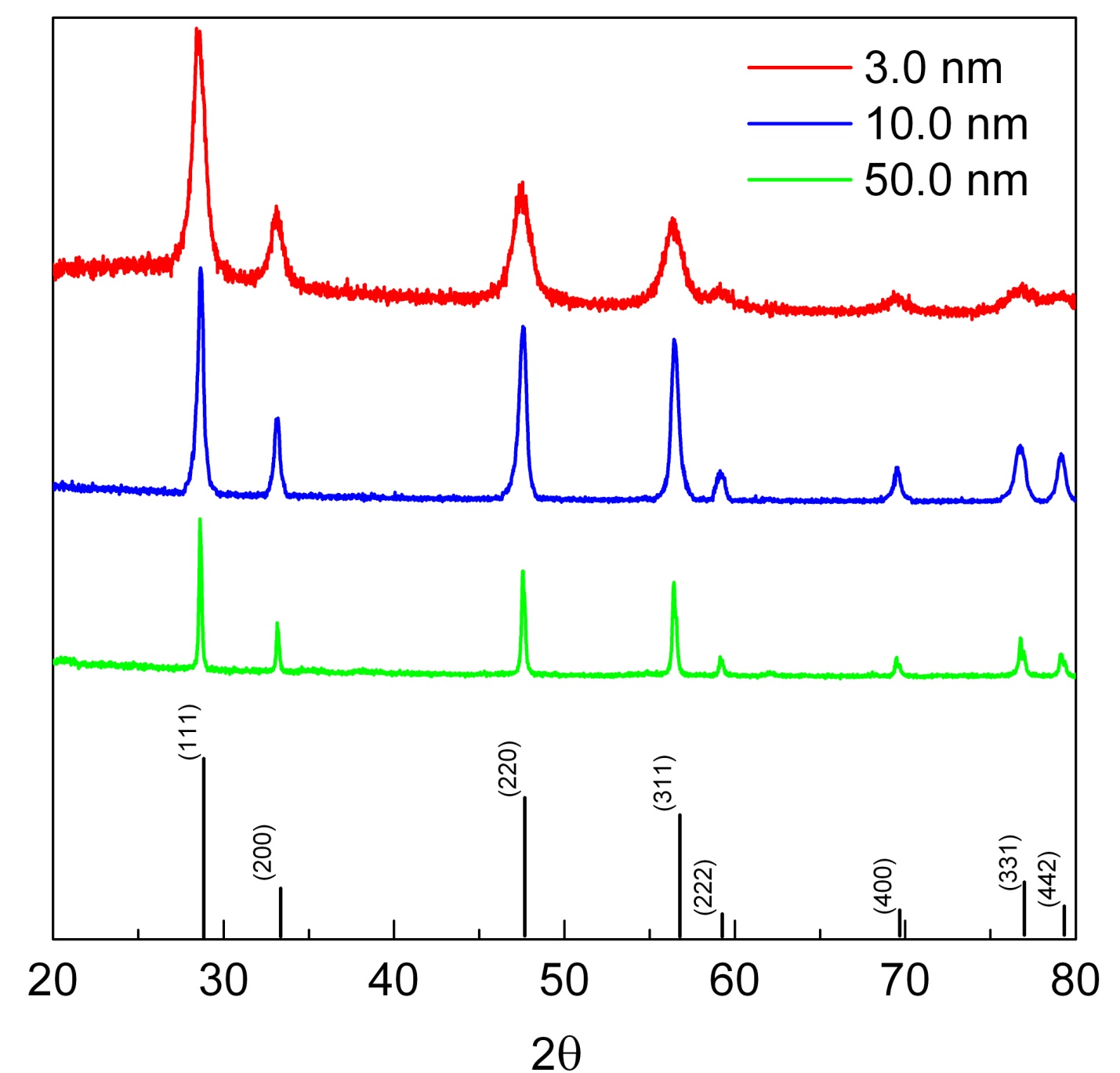
Figure S2.** XRD of tested nanoceria specimens: 3.0 nm nanoceria**,** 10.0 nm nanoceria and 50.0 nm nanoceria as obtained.

**
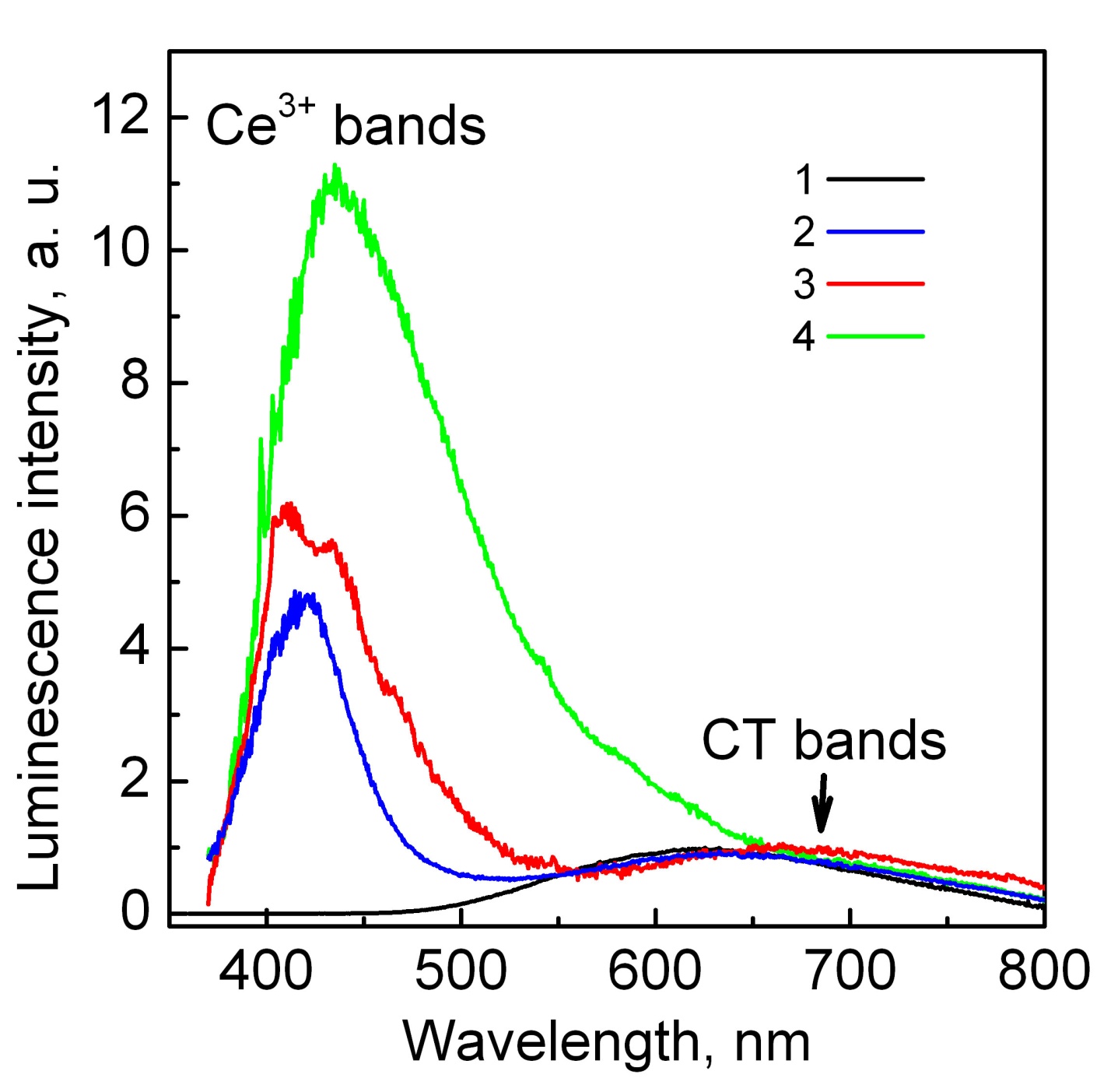
Figure S3.** The low temperature (77K) luminescence spectra of various specimens of nanoceria powders normalized to the CT band intensity for comparison of their $C_{V_{O}}$: 1- 50.0 nm nanoceria as obtained; 2 - Y^3+^ doped 50.0 nm nanoceria (The Ce^3+^ band intensity of Y^3+^ doped 50.0 nm nanoceria can be changed by varying the concentration of Y^3+^ ions); 3 – 10.0 nm nanoceria; 4 - 3.0 nm nanoceria.

**
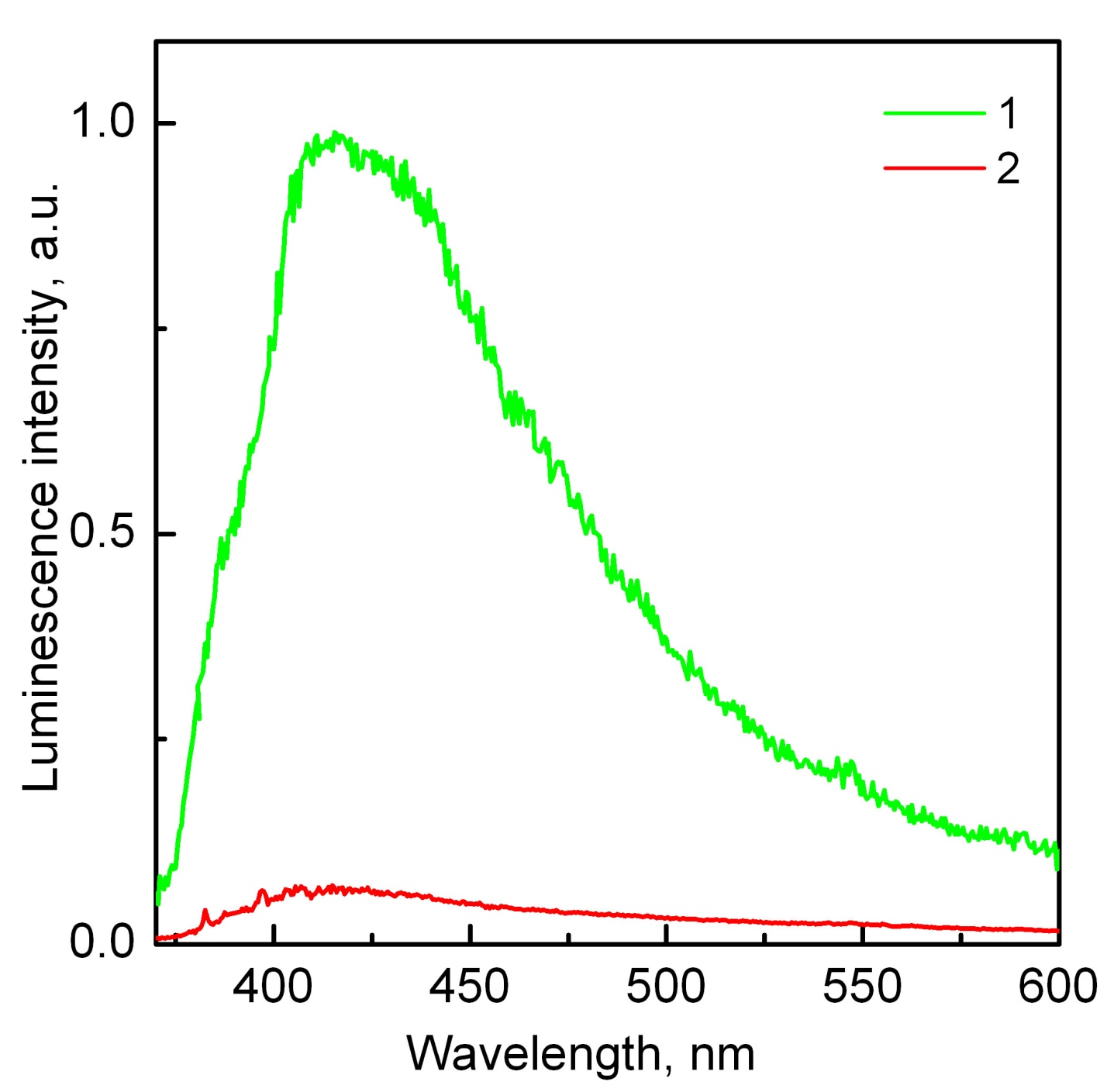
Figure S4.** The luminescence spectra of 10.0 nm nanoceria powder before (1) and after (2) annealing in oxygen atmosphere.

**
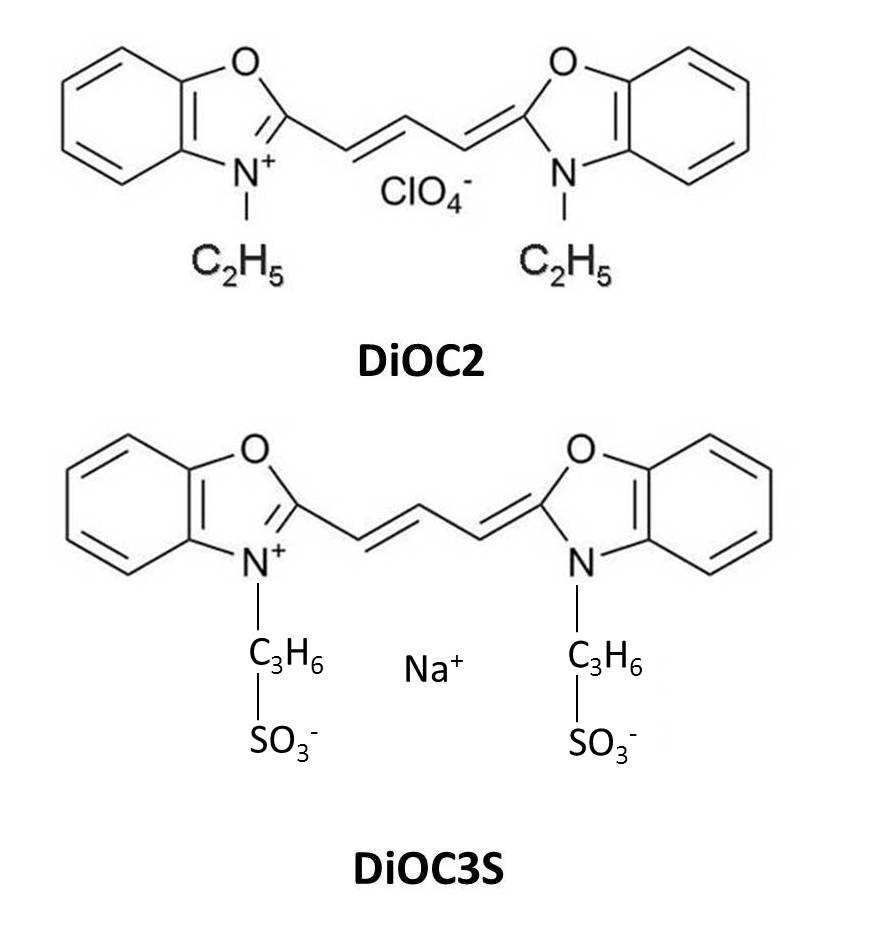
**

**Figure S5.** The structural formulas of DiOC2 (cationic) and DiOC3S (anionic) dyes.

**
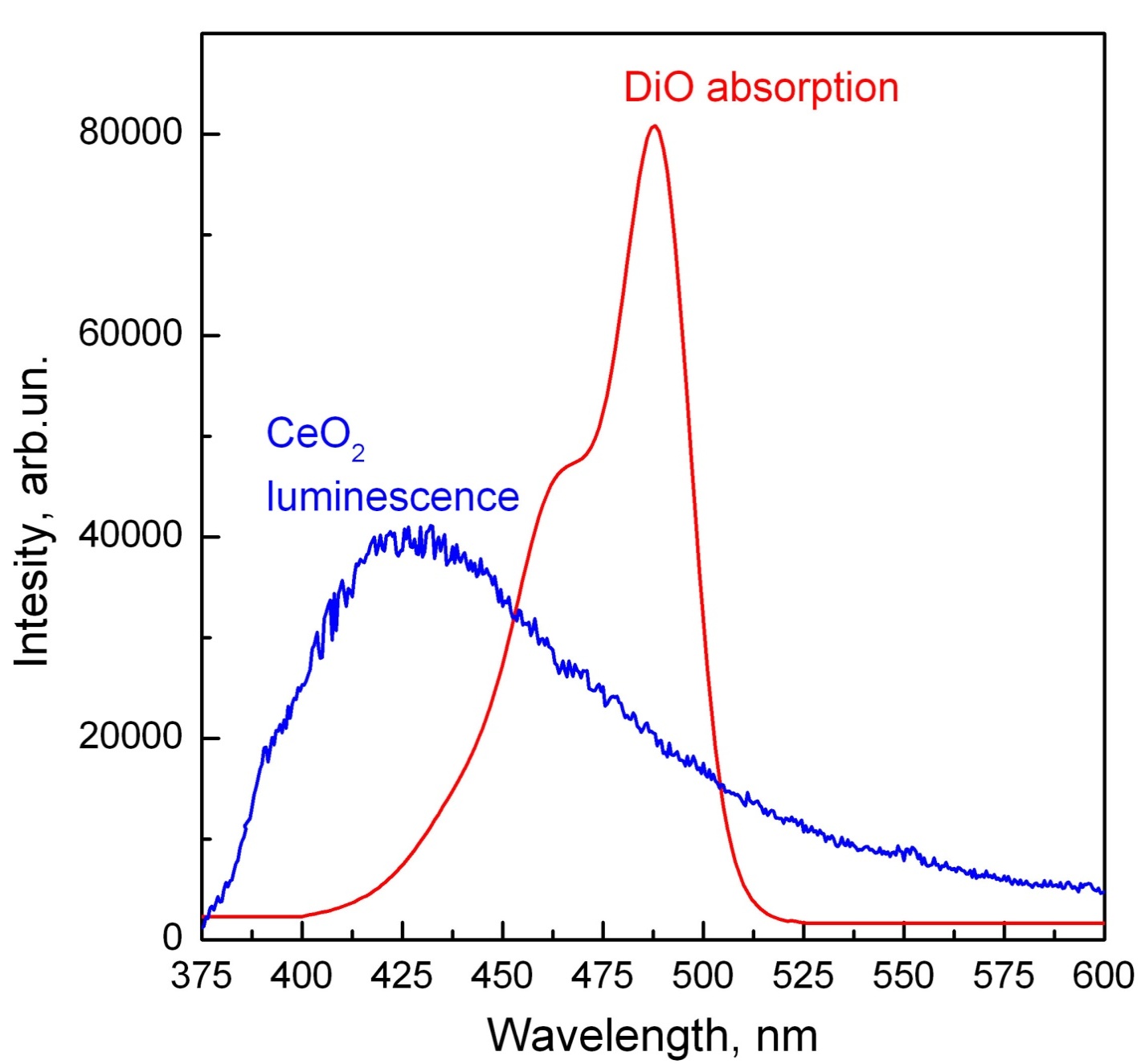
Figure S6.** The luminescence spectrum of 10.0 nm nanoceria and absorption spectrum of DiOC3S, which was similar to the spectrum of DiOC2 dye.

**
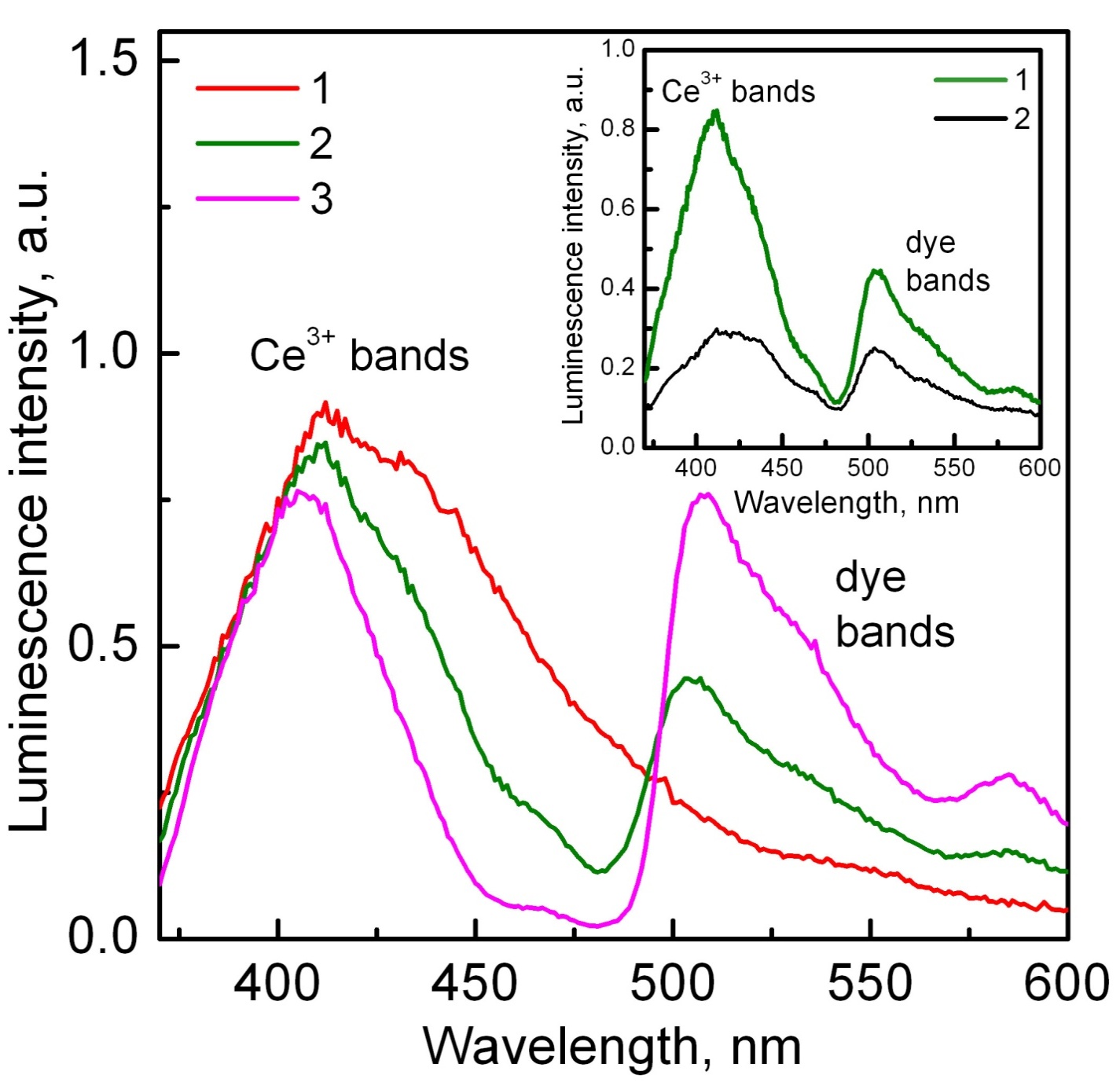
Figure S7.** Luminescence spectra of 10.0 nm nanoceria before (1) and after addition 50 μl (2) and 80 μl (3) of DiOC3S anionic dye, in the insert – luminescence spectra of 10.0 nm nanoceria after addition 50 μl of anionic dye (1) and subsequent addition of HP (C=0.1 mM) (2).

**
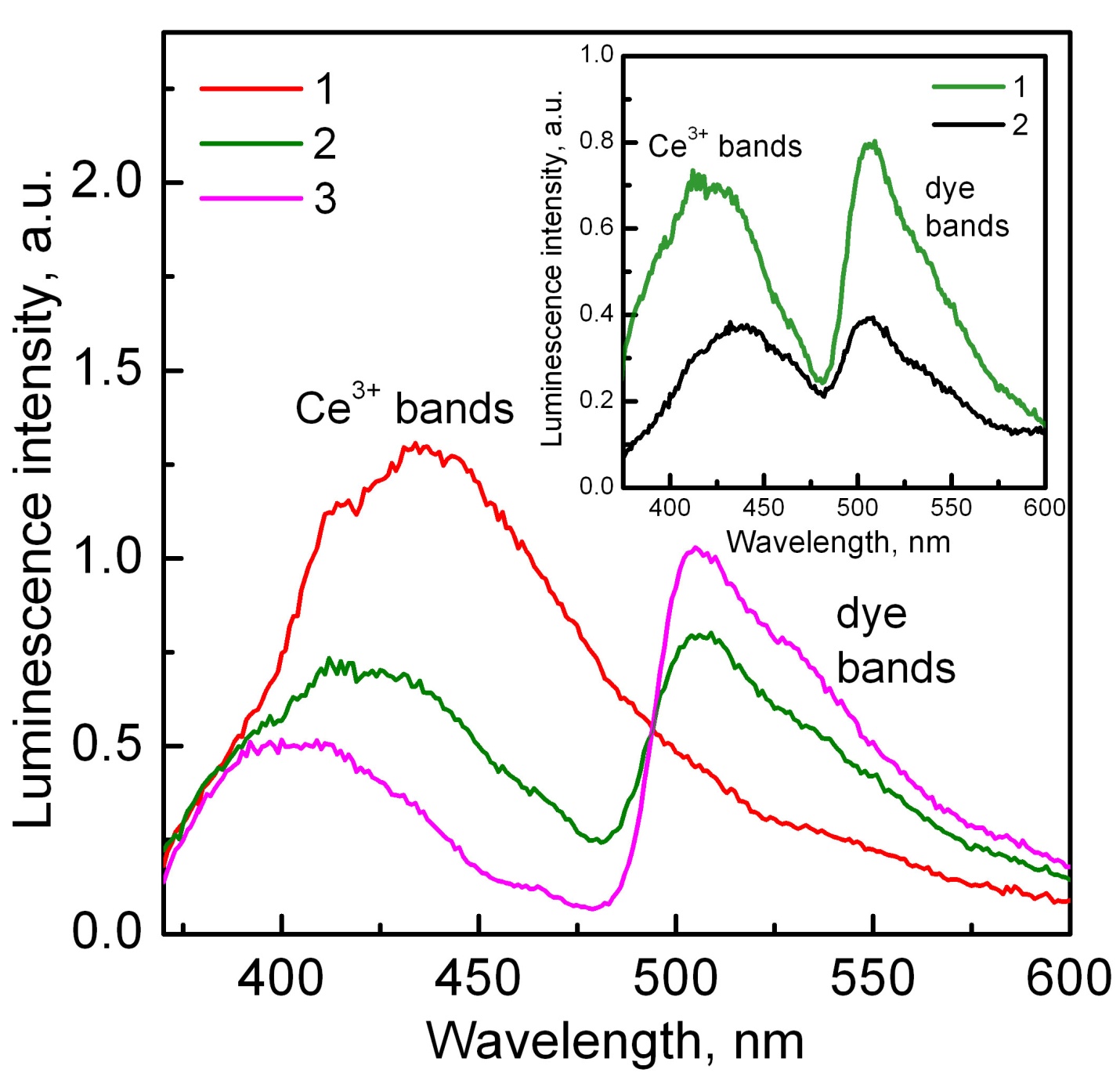
Figure S8.** The luminescence spectra of 10.0 nm nanoceria before (1) and after addition 50 μl (2) and 80 μl (3) of DiOC2 cationic dye, in the insert – luminescence spectra of 10.0 nm nanoceria after addition 50 μl of cationic dye (1) and subsequent addition of HP (C=0.1 mM) (2).
